# The incidence of hip, forearm, humeral, ankle, and vertebral fragility fractures in Italy: results from a 3-year multicenter study

**DOI:** 10.1186/ar3213

**Published:** 2010-12-29

**Authors:** Umberto Tarantino, Antonio Capone, Marco Planta, Michele D'Arienzo, Giulia Letizia Mauro, Angelo Impagliazzo, Alessandro Formica, Francesco Pallotta, Vittorio Patella, Antonio Spinarelli, Ugo Pazzaglia, Guido Zarattini, Mauro Roselli, Giuseppina Montanari, Giuseppe Sessa, Marco Privitera, Cesare Verdoia, Costantino Corradini, Maurizio Feola, Antonio Padolino, Luca Saturnino, Alessandro Scialdoni, Cecilia Rao, Giovanni Iolascon, Maria Luisa Brandi, Prisco Piscitelli

**Affiliations:** 1 Division of Orthopaedics and Traumatology, Tor Vergata Foundation University Hospital, University of Rome, Tor Vergata, Viale Oxford 81, Rome, 00133, Italy; 2 Department of Orthopaedics, University of Cagliari, Lungomare Poetto, Cagliari, 09124, Italy; 3 Division of Orthopaedic and Traumatology, University of Palermo, Via Antonio Veneziano 120, Palermo, 90139, Italy; 4 Department of Physical and Rehabilitative Medicine, University of Palermo, Via Antonio Veneziano 120, Palermo, 90139, Italy; 5 Division of Orthopaedics and Traumatology I, San Giovanni Addolarata Britannico Hospital, Via dell'Amba Aradam 9, Rome, 00184, Italy; 6 Division of Orthogeriatrics, San Camillo Hospital, Piazza Carlo Forlanini 1, Rome, 00151, Italy; 7 Division of Orthopaedics and Traumatology, University of Bari, Piazza Giulio Cesare 11, Bari, 70124, Italy; 8 Division of Orthopaedics and Traumatology II, Spedali Riuniti di Brescia, Piazzale Spedali Civili 1, Brescia, 25123, Italy; 9 Division of Orthopaedics and Traumatology, Maria Vittoria Hospital, Via Cibrario 72, Turin, 10144, Italy; 10 Division of Orthopaedics, University of Catania, University Hospital Vittorio Emanuele, Via S.Sofia 78, Catania, 95123, Italy; 11 Department of Orthopaedics and Traumatology I, University of Milan, Orthopedic Institute G.Pini, Piazza Cardinale Ferrari 1, Milan, 20100, Italy; 12 Department of Orthopaedics and Rehabilitative Medicine, Second University of Naples, Via Luigi De Crecchio, Naples, 80138, Italy; 13 Department of Internal Medicine, University of Florence, Viale Pieraccini 18 50134 Florence, Italy

## Abstract

**Introduction:**

We aimed to assess the incidence and hospitalization rate of hip and "minor" fragility fractures in the Italian population.

**Methods:**

We carried out a 3-year survey at 10 major Italian emergency departments to evaluate the hospitalization rate of hip, forearm, humeral, ankle, and vertebral fragility fractures in people 45 years or older between 2004 and 2006, both men and women. These data were compared with those recorded in the national hospitalizations database (SDO) to assess the overall incidence of fragility fractures occurring at hip and other sites, including also those events not resulting in hospital admissions.

**Results:**

We observed 29,017 fractures across 3 years, with hospitalization rates of 93.0% for hip fractures, 36.3% for humeral fractures, 31.3% for ankle fractures, 22.6% for forearm/wrist fractures, and 27.6% for clinical vertebral fractures. According to the analyses performed with the Italian hospitalization database in year 2006, we estimated an annual incidence of 87,000 hip, 48,000 humeral, 36,000 ankle, 85,000 wrist, and 155,000 vertebral fragility fractures in people aged 45 years or older (thus resulting in almost 410,000 new fractures per year). Clinical vertebral fractures were recorded in 47,000 events per year.

**Conclusions:**

The burden of fragility fractures in the Italian population is very high and calls for effective preventive strategies.

## Introduction

Italy has one of the highest life expectancies in the world: according to the Italian National Institute for Statistics (ISTAT), life expectancy at birth increased at a rate of 4 months per year from 1950 to 2005, reaching 78.4 years for men and 87.4 years for women, respectively [1,2]. Twenty percent of the Italian population (12,085,058 people) is actually older than 65 years [1], but 5.6% of these are 80 years and older [1]. The national aging index was recently computed at 143.1, with southern Italian regions younger than northern areas of the country [1]. Increased life expectancy is associated with a greater frailty of elderly people and a higher prevalence of chronic and degenerative diseases, including osteoporosis. The World Health Organization (WHO) considers osteoporosis to be second only to cardiovascular diseases as a critical health problem [3], and previous analyses have shown that the incidence and costs of hip fractures in Italy are already comparable to those of acute myocardial infarction [4]. The main Epidemiological Study on the Prevalence of Osteoporosis in Italy (ESOPO) reported a high prevalence of osteoporosis: 23% among all women, with age-specific rates ranging from 9% (40- to 49-year-olds) up to 45% (70 to 79 years or older), and almost 15% in men aged 60 years and older [5,6]. According to these data, about 4 million of Italian women and 800 thousand men are thought to be affected by osteoporosis [2]. However, an overestimation of these prevalence data cannot be excluded, as the ESOPO study was conducted by using QUS (quantitative ultrasound) measurements, given the lack of national epidemiologic studies performed by using DEXA (dual-energy x-ray absorptiometry), the gold-standard tool in the diagnosis of osteoporosis [6-8]. It is known that osteoporosis is a condition that enhances the risk of fractures [9], and osteoporotic fractures represent a challenge for health professionals and decision makers in the 21^st ^century. Despite these observations, only limited data are available about the incidence of fragility fractures in the Italian population [10-13], particularly concerning fractures occurred in skeletal sites other than hip. Vertebral fractures or deformities are the most common osteoporotic fractures [14]. According to the European Vertebral Osteoporosis Study (EVOS), in about 12% of both men and women aged 50 through 80 years, it is possible to detect vertebral deformities, with their prevalence increasing with age in both sexes [15]. Vertebral deformities, even if asymptomatic, are associated with adverse outcomes, including back pain, physical impairment [16,17], a higher risk of subsequent osteoporotic fractures [18-20], and an increased risk of mortality [19,21]. However, two thirds of vertebral fractures do not come to clinical attention [22], and it is very difficult to assess their incidence among the general population. Wrist or forearm fractures represent the most common breakage among perimenopausal women (typically between 40 and 50 years old), with their incidence increasing quickly after the menopause, probably as a consequence of a hormone-related fast bone-loss process, but reaching a plateau after the age of 65 [23]. Wrist fractures are also frequent in men younger than 70 years, but the age-adjusted female-to-male ratio remains 4:1 [23]. Wrist fractures increase almost twofold the risk of subsequent hip or vertebral fractures, but also the risk of new forearm breakage and other skeletal fractures is increased by 3.3 and 2.4 times, respectively [24]. Humeral fractures represent the third most common fracture in people aged 65 years and older and have been associated with a higher risk of subsequent hip fractures [25]. Actually, a proximal humeral fracture increases more than 5 times the risk of hip fracture at 1 year [25]. Incidence rates estimated for fractures of the proximal humerus and other skeletal sites increase with age and seem to be more frequent in women with poor neuromuscular function but also in aging men, with 75% of these fractures being caused by moderate- or low-energy trauma [23,26]. Even fractures occurring at foot/ankle or ribs have been found to double the risk of subsequent hip, vertebral, forearm, or other skeletal fractures [24], thus confirming that all osteoporotic fractures should be considered the first signal of an evolving disease. Our aim was to estimate the incidence and hospitalization rate of the most common fragility fractures in Italy: hip fractures, and, for the first time, other "minor" fractures such as forearm, humeral, ankle, and vertebral fractures, which do not result automatically in hospital admissions.

## Materials and methods

### Patients and survey

We carried out a survey of 29,017 fractured patients referring to the emergency departments of 10 major Italian hospitals in different Northern, Central, and Southern regions of the country. The hospitals involved in the survey were the following: Milan (Othopedic Institute "Gaetano Pini"), Turin (Maria Vittoria Hospital), Brescia (Riuniti Hospital), Rome (Tor Vergata University Hospital, St. Camillo Hospital and St. Giovanni Addolarata Hospital), Cagliari (University Hospital), Palermo (University Hospital), Bari (University Hospital), and Catania (University hospital). Orthopedic surgeons at each hospital were involved in this survey, because all kinds of fractures admitted to emergency departments were treated by orthopaedics departments. Physicians involved were asked systematically to record specific data concerning the fractures observed between 01/01/2004 and 31/12/2006: the skeletal site of the fracture, the gender and age of the patient, and the type of trauma of the patient (low-energy trauma or not). Fractures incurred because of low-energy trauma were considered osteoporotic fragility fractures. Orthopedic surgeons involved in the study recorded whether the patient was discharged from emergency department after having been treated or if the patient was hospitalized because of the fracture. The survey included hip, humeral, ankle, forearm, and vertebral fractures. Information obtained did not include demographic factors, osteoporotic status, tobacco and alcohol history, medications consumption, history of falls, fracture risk factors, or previous fracture history. The patient age was computed from date of birth. Participants were stratified into three age groups: 45 to 64, 65 to 74, and 75 years or older. Because the osteoporotic status of the participants was not instrumentally investigated (no data concerning bone mineral density were available) and to be more conservative, avoiding false-positive cases, men aged 45 to 64 years (*n *= 3,183) were always excluded from data analyses, even though their fractures were classified as due to low-energy trauma. Conversely, women in the same age group (45 to 64 years old; *n *= 5,501) were included only in the data analyses concerning humeral, ankle, wrist, and vertebral fractures (but not in the analysis of hip fragility fractures, which were computed only in those older than 65 years) because of the high prevalence of these kinds of "minor" fragility fractures in younger postmenopausal women [15,23]. For each kind of fracture, we computed the number of patients requiring hospital admission and the number of patients discharged directly from the emergency department after having been examined and treated (radiologic examination, orthopedic evaluation, and treatments not requiring hospitalization). The hospitalization rate (the percentage of patients requiring hospital admission versus the overall number of patients with a hip, wrist, humeral, ankle, or vertebral fracture) was computed for each kind of fracture. Population data concerning the 3 examined years were obtained from the Italian Institute for Statistics (ISTAT). Data were processed by using Stata (StataCorp, College Station, TX, USA) and Excel (Microsoft, Redmond, WA, USA) software.

### Comparative analysis

The second part of our study consisted of the analysis of the National Hospital Discharge records (SDO) maintained at the Italian Ministry of Health, concerning the 3 years of our survey (2004 through 2006). In this archive, information concerning all hospitalizations occurring in Italian public and private care settings are collected. These data are anonymous and include the patient's age, diagnosis, procedures performed, and length of the hospitalization. It is known that about 90% of hip fractures systematically result in hospitalization, thus allowing researchers to perform epidemiologic analyses by using hospital discharge records [13]. Conversely, only a small proportion of patients with osteoporotic fractures at other different skeletal sites are hospitalized [23], so that hospital discharge records cannot simply be used to investigate the prevalence of most osteoporotic fractures. In this perspective, we have used the hospitalization rates observed in the sample of our multicenter survey for each kind of fracture (humeral, ankle, forearm, hip, and vertebral fractures) to estimate the number of fracture patients discharged all over the country from emergency departments without being hospitalized. Descriptive statistical analyses were used to calculate the annual incidence of hip, humeral, ankle, forearm, hip, and vertebral fractures in the whole Italian population, by applying the hospitalization rates observed in our survey to the number of hospital admissions available at the national level for each kind of fracture across the 3 examined years. Because almost all patients with hip, humeral, ankle, or forearm fractures are referred to the hospital, whereas only a minority (from 22% to 33%) of vertebral fractures, defined as "clinical vertebral fractures," come to medical attention [27-30], we had to perform a corrective analysis to estimate the incidence rate of vertebral fractures in the whole Italian population. The hospitalization rate computed in our survey concerning vertebral fractures included only patients referring to the hospital because of clinical vertebral fractures, whereas the majority of vertebral deformities (from 78% to 67%) are asymptomatic and do not require admission at emergency departments [27-30]. To be conservative, we considered 70% of vertebral deformities occurring in Italy to be asymptomatic, and 30% of them as "clinical" fractures. Therefore, we took into account this proportion when performing comparative analyses between the hospitalization rate computed for vertebral fractures in our survey and data from the National Hospitalization Database (SDO). To acquire all the necessary data concerning hospitalizations, the SDO archive was enquired for the following ICD-9CM diagnosis codes (limited to major diagnosis): 820.0 to 820.1 (femoral neck fractures), 820.2 to 820.3 (per-trochanteric femoral fractures), 820.8, 820.9, 821.1 (other femoral fractures), 812 (humeral fractures), 824 (ankle fractures), 813 (forearm/wrist fractures), and 805 (vertebral fractures). Data were stratified by gender and into three age groups (65 to 74 years and 75 years and older) and were processed by using Stata (StataCorp) and Excel (Microsoft) software.

## Results

An overall number of 29,017 patients with fractures were enrolled over a period of 3 years. Table 1 shows the composition of the population involved in the survey per each selected age group and the distribution per age group and gender of the enrolled patients, both those discharged from Emergency Department and those hospitalized after any fracture considered in the protocol. Tables 2, 3, 4, 5, and 6 show the yearly number of hospitalizations after hip, humeral, forearm/wrist, ankle, and vertebral fractures recorded during the study period in the Italian National Hospital Discharge records (SDO 2004-2005-2006). About 70% of the overall fractures observed during the study period (*n *= 20,333) occurred in persons aged older than 65 years. In total, 25,495 were classified by clinicians as fragility fractures (a consequence of low-energy trauma), whereas 3,522 events were regarded as fractures induced by high-energy trauma, mostly affecting men aged 45 through 64 years old (*n *= 3,183). We recorded a total of 8,290 hip fragility fractures (1,974 men and 6,316 women), 4,559 humeral fragility fractures (976 men and 3,583 women), 2,981 ankle fragility fractures (494 men and 2,487 women), 6,514 forearm/wrist fragility fractures (786 men and 5,728 women), and 2,927 vertebral fragility fractures (577 men and 2,350 women). Hospitalization rates were the following: 93.0% for hip fractures (*n *= 7,711), 36.3% for humeral fractures (*n *= 1,657), 31.3% for ankle fractures (*n *= 932), 22.6% for forearm/wrist fractures (*n *= 1,475), and 27.6% for clinical vertebral fractures (*n *= 809). Conversely, emergency departments directly discharged 7.0% of hip fracture patients (*n *= 579), 63.7% of humeral fractures (*n *= 2,902), 68.7% of ankle fractures (*n *= 2,049), 77.4% of forearm/wrist fractures (*n *= 5,039), and 72.4% of vertebral fractures (*n *= 2,118). Women accounted for 49.0% of the overall hospitalizations and for 51.0% of total discharges from the emergency departments. Tables 7, 8, 9, 10, and 11 list the number of patients hospitalized or discharged from the emergency department after hip, humeral, ankle, forearm/wrist, and vertebral fractures per gender and age group. According to the analyses performed on the National Hospitalizations Database, the overall number of hip and other "minor" fragility fractures occurring each year in Italy has been estimated at almost 410,000 events. The annual incidence of the overall most common fragility fractures (hip, wrist, vertebral, humeral, and ankle fractures) per 100 inhabitants has been estimated up to 1.53 in men aged older than 65 years and up to 3.94 in women of the same age group. The incidence per 100 inhabitants reached 2.35 and 4.67 in men and women aged older than 75 years, respectively (with women aged older than 75 years the age group in which the highest number of fragility fractures was detected). Table 12 summarizes the incidence of fragility fractures per 100 inhabitants in year 2006 (according to gender and overall). Specifically, we estimated for the year 2006 (Table 13) an annual incidence of about 87,000 hip fragility fractures (corresponding to an incidence rate of 0.75 per 100 people older than 65 years: 0.41 for men and up to 1.0 for women), 48,000 humeral fragility fractures (0.16 for men older than 64 years and 0.28 for women older than 45 years), 36,000 ankle fragility fractures (0.19 per 100 adults aged >45: 0.11 for men and 0.22 for women), 85,000 forearm/wrist fragility fractures (0.44 per 100 adults older than 45 years: 0.15 for men and up to 0.55 for women), and 155,000 vertebral fractures (0.24 per 100 adults older than 45 years: 0.22 for men and up to 0.25 for women). Clinical vertebral fractures were estimated at 47,000 events per year (0.24 per 100 adults 45 years and older: 0.22 for men and up to 0.25 for women), and were assumed to represent almost 30% of the overall incident vertebral fractures [22,27-30]. The ratio of female-to-male patients (F/M ratio) for each kind of fracture always showed positive values in favor of women, with an increasing trend from the youngest to the oldest age group (Table 13). The highest F/M ratio (9.04) was observed for wrist fractures in people aged 65 years and older (5.09 in people aged 65 to 74 years and 9.04 in those older than 75 years). Humeral fractures showed an F/M ratio of 4.10 for people older than 65 years (2.99 in people aged 65 through 74 years old, and 4.98 for those older than 75 years). The F/M ratio for hip fractures was 3.43 for people older than 65 (2.48 in people aged T F/M ratio for all vertebral fractures was 2.64 over 65 of age, 2.01 in people aged between 65 and 74 years, and 3.27 in subjects older than 75 years.

**Table 1:** Study sample: enrolled patients distributed per age group, gender, and hospitalization status

Age group (years)		Males		Females		Total (M + F)	
				
		ER Not hospitalized	Hospitalized	ER Not hospitalized	Hospitalized	ER Not hospitalized	Hospitalized
45 to 64		1,812	1,371	3,575	1,926	5,387	3,297
		3,183		5,501		8,684	

65 to 74		988	901	2,911	2,138	3,899	3,039
		1,889		5,049		6,938	

Older than 75		1,047	1,871	4,235	6,242	5,282	8,113
		2,918		10,477		13,395	

Total	ER/H	3,847	4,143	10,721	10,306	14,568	14,449
	ER + H	7,990		21,027		29,017	

ER, patients referring to Emergency Room.

**Table 2:** Yearly number of hospitalizations after hip fractures recorded in the National Hospital Discharge records (SDO, 2004, 2005, 2006) maintained at the Italian Ministry of Health

	2004			2005			2006		
			
Age (years)	M	F	Subtotal	M	F	Subtotal	M	F	Subtotal
45 to 64	2,979	3,810	6,789	2,961	3,632	6,593	3,002	3,804	6,806
65 to 74	3,813	9,430	13,243	3,660	9,352	13,012	3,765	9,322	13,087
Older than 75	12,958	49,589	62,547	13,937	52,051	65,988	14,593	53,259	67,852
Total	19,750	62,829	82,579	20,558	65,035	85,593	21,360	66,385	87,745

These data exclude hospital readmissions of the same patients.

**Table 3:** Yearly number of hospitalizations after humeral fractures recorded in the National Hospital Discharge records (SDO, 2004-2005-2006) maintained at the Italian Ministry of Health

	2004			2005			2006		
			
Age (years)	M	F	Subtotal	M	F	Subtotal	M	F	Subtotal
45 to 64	1,994	3,159	5,153	2,005	3,323	5,328	2,123	3,355	5,478
65 to 74	1,026	4,247	5,273	1,099	4,240	5,339	1,138	4,311	5,449
Older than 75	1,370	6,949	8,319	1,437	7,077	8,514	1,425	7,452	8,877
Total	4,390	14,355	18,745	4,541	14,640	19,181	4,686	15,118	19,804

These data exclude hospital readmissions of the same patients.

**Table 4:** Yearly number of hospitalizations after forearm/wrist fractures recorded in the National Hospital Discharge records (SDO, 2004-2005-2006) maintained at the Italian Ministry of Health

Age (years)	2004			2005			2006		
			
	M	F	Subtotal	M	F	Subtotal	M	F	Subtotal
45 to 64	3,808	6,270	10,078	3,886	6,308	10,194	4,029	6,610	10,639
65 to 74	1,227	5,125	6,352	1,241	5,160	6,401	1,209	5,036	6,245
Older than 75	826	5,322	6,148	875	5,461	6,336	872	5,550	6,422
Total	5,861	16,717	22,578	6,002	16,929	22,931	6,110	17,196	23,306

These data exclude hospital readmissions of the same patients.

**Table 5:** Yearly number of hospitalizations after ankle fractures recorded in the National Hospital Discharge records (SDO, 2004-2005-2006) maintained at the Italian Ministry of Health

	2004			2005			2006		
			
Age (years)	M	F	Subtotal	M	F	Subtotal	M	F	SUBTOTAL
45 to 64	3,177	5,106	8,283	3,125	5,025	8,150	3,344	4,919	8,263
65 to 74	1,187	2,778	3,965	1,213	2,732	3,945	1,236	2,839	4,075
Older than 75	633	1,728	2,361	681	1,777	2,458	721	1,765	2,486
Total	4,997	9,612	14,609	5,019	9,534	14,553	5,301	9,523	14,824

**Table 6:** Yearly number of hospitalizations after vertebral fractures recorded in the National Hospital Discharge records (SDO, 2004-2005-2006) maintained at the Italian Ministry of Health

	2004			2005			2006		
			
Age (years)	M	F	Subtotal	M	F	Subtotal	M	F	Subtotal
45 to 64	3,079	2,678	5,757	2,998	2,614	5,612	3,021	2,667	5,688
65 to 74	1,735	2,560	4,295	1,821	2,557	4,378	1,891	2,583	4,474
Older than 75	1,644	3,812	5,456	1,697	3,841	5,538	1,832	3,942	5,774
Total	6,458	9,050	15,508	6,516	9,012	15,528	6,744	9,192	15,936

These data exclude hospital readmissions of the same patients.

**Table 7:** Hip fragility fractures

M	F	Subtotal patients hospitalized	Age group (years)	M	F	Subtotal patients not hospitalized
412	951	1,363	**65 to 74**	37	88	125
1,433	4,915	6,348	**Older than 75**	92	362	454
1,845	5,866	7,711	**Total**	129	450	579

Patients hospitalized		93.0%		Patients not hospitalized		7.0%

Number of patients hospitalized because of the fracture versus number of patients discharged directly from the Emergency Department.

**Table 8:** Humeral fragility fractures

M	F	Subtotal patients hospitalized	Age group (years)	M	F	Subtotal patients not hospitalized
	332	332	**45 to 64**		473	473
136	405	541	**65 to 74**	284	581	865
179	605	784	**Older than 75**	377	1,187	1,564
315	1,342	1,657	**Total**	661	2,241	2,902

Patients hospitalized		36.3%		Patients not hospitalized		63.7%

Number of patients hospitalized because of the fracture versus number of patients discharged directly from the Emergency Department.

**Table 9:** Ankle fragility fractures

M	F	Subtotal patients hospitalized	Age group (years)	M	F	Subtotal patients not hospitalized
	377	377	**45 to 64**		657	657
119	212	331	**65 to 74**	185	531	716
58	166	224	**Older than 75**	132	544	676
177	755	932	**Total**	317	1,732	2,049

Patients hospitalized		31.3%		Patients not hospitalized		68.7%

Number of patients hospitalized because of the fracture versus number of patients discharged directly from the Emergency Department.

**Table 10:** Forearm fragility fractures

M	F	Subtotal patients hospitalized	Age group (years)	M	F	Subtotal patients not hospitalized
	478	478	**45 to 64**		1,814	1,814
139	415	554	**65 to 74**	311	1,228	1,539
93	350	443	**Older than 75**	243	1,443	1,686
232	1,243	1,475	**Total**	554	4,485	5,039

Patients hospitalized		22.6%		Patients not hospitalized		77.4%

Number of patients hospitalized because of the fracture versus number of patients discharged directly from the Emergency Department.

**Table 11:** Vertebral fragility fractures

M	F	Subtotal patients hospitalized	Age group (years)	M	F	Subtotal patients not hospitalized
	245	245	**45 to 64**		562	562
95	155	250	**65 to 74**	171	483	654
108	206	314	**Older than 75**	203	699	902
203	606	809	**Total**	374	1,744	2,118

Patients hospitalized		27.6%		Patients not hospitalized		72.4%

Number of patients hospitalized because of the fracture versus number of patients discharged directly from the Emergency Department.

**Table 12:** Incidence of fragility fractures per 100 inhabitants in Italy (2006)

Fractures	M	F	Total
Hip (M > 65 + F > 65)	0.41	1.0	0.75
Humerus (M > 65 + F > 65)	0.16	0.28	0.25
Ankle (M > 65 + F > 65)	0.11	0.22	0.19
Wrist (M > 65 + F > 65)	0.15	0.55	0.44
Vertebra clinical fractures (M > 65 + F > 65)	0.22	0.25	0.24

**Table 13:** Overall estimation of fragility fractures and F/M ratio in Italy (2006)

	Total	F/M Ratio in patients older than 65 years		
		
		65 to 74 years	Older than 75 years	Overall older than 65 years
Hip fractures (M > 65 + F > 65)	87,000	2.48	3.68	3.43
Humeral fractures (M > 65 + F > 45)	48,000	2.99	4.98	4.10
Ankle fractures (M > 65 + F > 45)	36,000	3.15	3.19	3.17
Wrist fractures (M > 65 + F > 45)	85,000	5.01	9.04	6.85
Vertebral fractures	Clinical fractures 47,000	2.01	3.27	2.64
(M > 65 + F > 45)	Overall fractures 155,000			

## Discussion

Hip fractures in Italy represent a serious health problem, and our estimations are consistent with other figures reported in previous national studies [4,13], which have estimated an increasing trend in the number of hospitalizations after hip fractures in Italy up to 94,000 admissions in the year 2005 (corresponding to about 85,000 individual patients). Conversely, fragility fractures occurring at skeletal sites other than the hip are an underestimated issue that is difficult to analyze because they do not systematically result in hospital admissions as a consequence of the lack of specific diagnostic codes for fragility fractures. While confirming the extremely high burden of hip fractures in the Italian population [13], at the same time, this study represents the first attempt to evaluate the incidence of "minor" fragility fractures in Italy. Until now, it was possible to refer to US, UK, Australian and Swedish data concerning fragility fractures other than those occurring at the hip [31-34]. According to these studies, the lifetime risk (percentage) of developing a vertebral clinical fracture or a forearm fracture in the United States at the age of 50 years has been estimated to be 15.6% and 16% in women or 5% and 2.5% in men, respectively [31]. The corresponding figures are 15.1% and 20.8% (women) or 8.3% and 4.6% (men), respectively, for clinical vertebral fractures and forearm fractures in Sweden [32], 3.1% and 16.6% (women) or 1.2% and 2.9% (men) in the UK [33], and 9.6% (spine) and 13.3% (wrist) in Australian women (no data available for men) [34]. However, it is difficult to use these rates in the evaluation of fracture incidence in the Italian population because the weight of people aged older than 65 years (ratio between elderly people and general population) is much higher in Italy than in the United States, Australia, or other European countries. These first Italian data, resulting from a 3-year multicenter clinical survey, could allow us to overcome the limitations arising from the use of foreign rates and are particularly valuable because Italy represents one of the countries with the highest life expectancies in the world, thus anticipating possible demographic scenarios of other European industrialized countries. Although the main limitation of the study is that it was not possible to analyze all fragility fractures occurring in Italy (as a consequence of the lack of a specific codification for fragility fractures and because only hospitalized fractures are recorded in national databases), our sample was likely to be representative of the whole Italian population who develop osteoporotic fragility fractures, thanks to the huge number of patients enrolled (29,017 with fractures), their distribution across the three different selected age groups (45 to 64, 65 to 74, and older than 75 years), and taking into account that the survey involved big hospitals of different Italian regions, thus overcoming possible interregional variability.

We are concerned about potential underestimation of vertebral fractures in our analysis because we have considered all clinical fractures to be referred to the Emergency Department, whereas in daily clinical practice, patients may also ask their general practitioners for a treatment or undergo a clinical evaluation while ambulatory. Conversely, we tried to avoid possible overestimations in the number of osteoporotic fractures by excluding from the analysis all men aged 45 to 64 years, even if investigators had classified those events as fragility fractures. Our data show that the absence of ICD9-CM codes for fragility fractures results in underestimation of "minor fractures" (those occurring at skeletal sites other than the hip), causing problems in the full evaluation of the osteoporosis impact in elderly people. Moreover, the underestimation of fragility fractures is also due to an underdiagnosis of osteoporosis in patients at higher risk (particularly postmenopausal women), resulting in undertreatment of this pathology and consequently in additional increase of osteoporotic fractures. On the contrary, it is known that appropriate treatments can prevent many osteoporotic fractures occurring in a high-risk population. Our data confirm an underestimation of "minor" fragility fractures and call for specific preventive strategies based on actions (such as optimization of access to antifracture therapies and compliance with the treatments, proper dietary calcium intake during the whole life, vitamin D supplementations, physical activity programs) to be carried out at the regional level all over the nation, as stated in the conclusions of the official inquiry promoted by the Italian Senate in 2002, specifically addressing the burden of osteoporosis in Italy [35]. Our data also emphasize the need for implementing a national registry of fragility fractures, whose start-up phase has been anticipated by this multicenter survey performed at Emergency Departments. The incidence rates resulting from this study may also be useful for carrying out further studies aimed to update national data of the Italian version of the international algorithm FRAX, which has been developed to provide physicians with a specific tool for the estimation of patients' individual risk of fragility fractures (as the algorithm is mainly based on data obtained from Scandinavian and North American populations) [36].

## Conclusions

Based on a 3-year multicenter survey, we have estimated in Italy an annual incidence of 410,000 new hip, humeral, wrist, ankle, and vertebral fragility fractures. These results confirm that osteoporosis is a leading cause of morbidity in the Italian population and a challenging health problem to be addressed by implementing appropriate preventive strategies.

## Abbreviations

ESOPO: Epidemiological Study on the Prevalence of Osteoporosis in Italy; EVOS: European Vertebral Osteoporosis Study; ISTAT: Italian National Institute for Statistics; SDO: National hospitalizations database; WHO: World Health Organization.

## Competing interests

UT, AC, MP, MDA, GLM, AI, AF, FP, VP, AS, UEP, GZ, MR, GM, GS, MP, CAV, CC, GI, and MLB have received research grants and funding for consulting/speaking from Merck, Chiesi, Sanofi-Aventis, Novartis, Stroder, Servier, Ely Lilly, Roche, and Nicomed; PP has received funding for consulting/speaking from Novartis, AMGEN, and Sanofi-Aventis; MF, AP, LS, AS, and CR have no disclosures.

## Authors' contributions

UT, AC, MP, MDA, GLM, AI, AF, FP, VP, AS, UEP, GZ, MR, GM, GS, MP, CAV, and CC conceived of the study, participated in its design, and assisted in the enrollment of all the patients in the study at each clinical center. UT coordinated the study. UT, PP, MF, AP, LS, AS, CR GI, and MLB performed all the descriptive and statistical analyses of the study and designed the outline of the article. All the authors contributed to drafting the manuscript.
